# Cannabidiol-loaded microparticles embedded in a porous hydrogel matrix for biomedical applications

**DOI:** 10.1007/s10856-023-06773-9

**Published:** 2024-02-14

**Authors:** Carla David, Jaqueline F. de Souza, Adriana F. Silva, Guillermo Grazioli, Andressa S. Barboza, Rafael G. Lund, André R. Fajardo, Rafael R. Moraes

**Affiliations:** 1 Biopathological Research Group, Faculty of Dentistry (GIBFO), University of the Andes, Mérida, Venezuela; 2 Graduate Program in Dentistry, Universidade Federal de Pelotas, Pelotas, Brazil; 3 Laboratory of Technology and Development of Composites and Polymeric Materials—LaCoPol, Universidade Federal de Pelotas, Pelotas, Brazil; 4 Department of Dental Materials, Universidad de la República, Montevideo, Uruguay

**Keywords:** Phytocannabinoids, Scaffolds, Chondroitin sulfate, Polyvinyl alcohol, Composite materials, Biomaterials

## Abstract

In this study, poly (lactic-co-glycolic acid) (PLGA) microparticles loaded with cannabidiol (CBD) were synthesized (PLGA@CBD microparticles) and embedded up to 10 wt% in a chondroitin sulfate/polyvinyl alcohol hydrogel matrix. In vitro chemical, physical, and biological assays were carried out to validate the potential use of the modified hydrogels as biomaterials. The microparticles had spherical morphology and a narrow range of size distribution. CBD encapsulation efficiency was around 52%, loading was approximately 50%. Microparticle addition to the hydrogels caused minor changes in their morphology, FTIR and thermal analyses confirmed these changes. Swelling degree and total porosity were reduced in the presence of microparticles, but similar hydrophilic and degradation in phosphate buffer solution behaviors were observed by all hydrogels. Rupture force and maximum strain at rupture were higher in the modified hydrogels, whereas modulus of elasticity was similar across all materials. Viability of primary human dental pulp cells up to 21 days was generally not influenced by the addition of PLGA@CBD microparticles. The control hydrogel showed no antimicrobial activity against *Staphylococcus aureus*, whereas hydrogels with 5% and 10% PLGA@CBD microparticles showed inhibition zones. In conclusion, the PLGA@CBD microparticles were fabricated and successfully embedded in a hydrogel matrix. Despite the hydrophobic nature of CBD, the physicochemical and morphological properties were generally similar for the hydrogels with and without the CBD-loaded microparticles. The data reported in this study suggested that this original biomaterial loaded with CBD oil has characteristics that could enable it to be used as a scaffold for tissue/cellular regeneration.

**Graphical Abstract:**

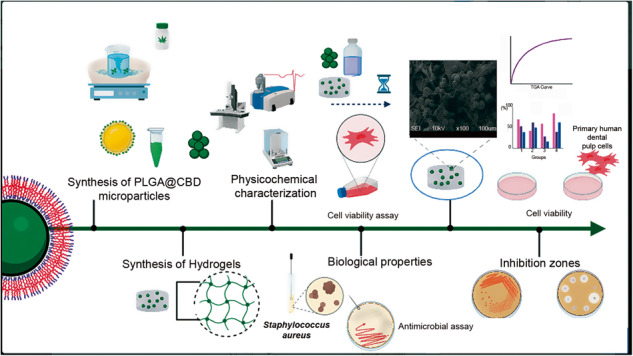

## Introduction

In tissue engineering, several biomaterials are used to manufacture scaffolds, including a variety of polymers, ceramics, metals, and biocompatible composites [1]. The scaffolds are engineered to provide, support, and induce bone or soft tissue regeneration [1, 2]. Overall, the three-dimensional structure of these materials offers mechanical resistance and allows the transport and delivery of drugs loaded in capsules or particles embedded within them [1, 3]. The capsules and particles may be vehicles for active molecules, cells, and nutrients acting as coadjuvants in the healing, repair, and remodeling processes of tissues [1, 4]. Specifically, polymeric microparticles are gaining interest not only as drug delivery systems but also as catalysts in tissue engineering [5–7].

Nowadays, there is a growing interest in the use and therapeutic potential of cannabidiol (CBD). This substance is one of more than 120 phytocannabinoids derived from the *Cannabis sativa* plant [8–11] with a chemical composition similar to that of delta-9-tetrahydrocannabinol (THC), but CBD is not psychotropic [8, 11]. The global CBD oil extract market was valued at US$ 7.94 billion in 2021 with a prospect of reaching US$ 37.74 billion in 2029 [12]. The increasing popularity of CBD may be attributed to its multiple medicinal properties, all of which have not yet been proven, including antiemetic, antioxidant [13–16], anticancer [9, 17, 18], regenerative [10, 19, 20], and anti-inflammatory [21] effects. CBD has been tested in therapies for cancer, epilepsy, pain, inflammation, and psychiatric disorders [8]. Furthermore, evidence suggests that CBD has antimicrobial [22, 23], biological, and osteoinductive properties with numerous potential biomedical applications [7, 20, 23–26].

Studies have tested CBD in drug delivery and release systems incorporated into scaffolds [20, 23, 25] due to its ability to interact directly or indirectly by potentiating all elements of the endocannabinoid system (CB1, CB2 receptors, and endogenous ligands). Cannabinoid-like actions may mediate tissue healing processes by recruiting regenerative cells at injury sites [10, 20, 23, 27]. In addition, CBD has the ability to stimulate undifferentiated mesenchymal cells by activation of the mitogen-activated protein kinases (plasma P42/44 MAPK receptors) and induce new cell lineages, such as osteoblasts [27–29]. CBD has shown positive in vitro effects on cell migration, collagen synthesis, mineral deposits, inflammatory response, and angiogenic activities [23].

The development of CBD formulations is challenging because the oleaginous nature of this substance endows it with high lipophilicity, hydrophobicity, and permeability [30–32]. CBD also has low stability and can be degraded by light- or auto-oxidation at room temperature [30]. Thus, effective loading and incorporation of CBD into compatible polymer-based vehicles, with improved aqueous solubility, could favor its positive properties and effects when added to biomaterials [22, 23, 30]. To overcome these limitations and improve the applicability of CBD for biomedical uses, in this study, we report the synthesis of microparticles of poly (lactic-co-glycolic acid) (PLGA), which served as reservoirs for the oil. The CBD-loaded microparticles were embedded in a hydrogel matrix of chondroitin sulfate (CS) and polyvinyl alcohol (PVA). Previously, our group demonstrated that CS/PVA hydrogels have remarkable properties that potentize their use as scaffolds [33]. The porous structure of these hydrogels offers support for the CBD-loaded microparticles by increasing their handling, which in turn facilitates the application of specific treatment sites. In addition to reporting the synthesis and characterization of the hydrogels embedded with the CBD-loaded microparticles, in vitro biological assays were also carried out to validate their potential use as biomaterials.

## Materials and methods

### Study design and reagents used

This in vitro study was designed to synthesize and characterize CBD-loaded PLGA microparticles (labeled herein as PLGA@CBD) that were later embedded within a hydrogel synthesized by using CS and PVA [2, 33]. The study was approved by the Institutional Review Board, Universidade Federal de Pelotas, Brazil (protocol 2.078.368). The reagents used were CBD (Epifractan; 5% CBD and less than 0.1% THC, Medic Plast SA, Montevideo, Uruguay), PLGA (65:35 ratio, Sigma-Aldrich, St. Louis, MI, USA), PVA (molar mass 85–124 kg/mol, 99% hydrolyzed, Sigma-Aldrich), CS (cat. 27042, ≥90%, Sigma-Aldrich), Tween 80 (Sigma-Aldrich), and dichloromethane (DCM, Synth, São Jose dos Campos, SP, Brazil). CBD was analyzed in a gas chromatograph coupled to a mass spectrophotometer (GC-MS; QP2010, Shimadzu Ultra, Kyoto, Japan). The presence of CBD in the commercial oil was confirmed by the presence of a higher load-mass band of CBD, as shown in the Supplemental material (S1). A capillary column (30 m × 0.32 mm × 0.25 µm) coated with 100% polydimethylsiloxane (Restek Corporation, Bellefonte, PA, USA) was used. An aliquot of the specimens was diluted in hexane (1:10) and 10 µL were injected at 200 °C in split mode. The oven temperature was set to 60 °C for 5 min and increased to 180 °C for an additional 5 min.

### Synthesis of PLGA@CBD microparticles

**Fig. 1:**
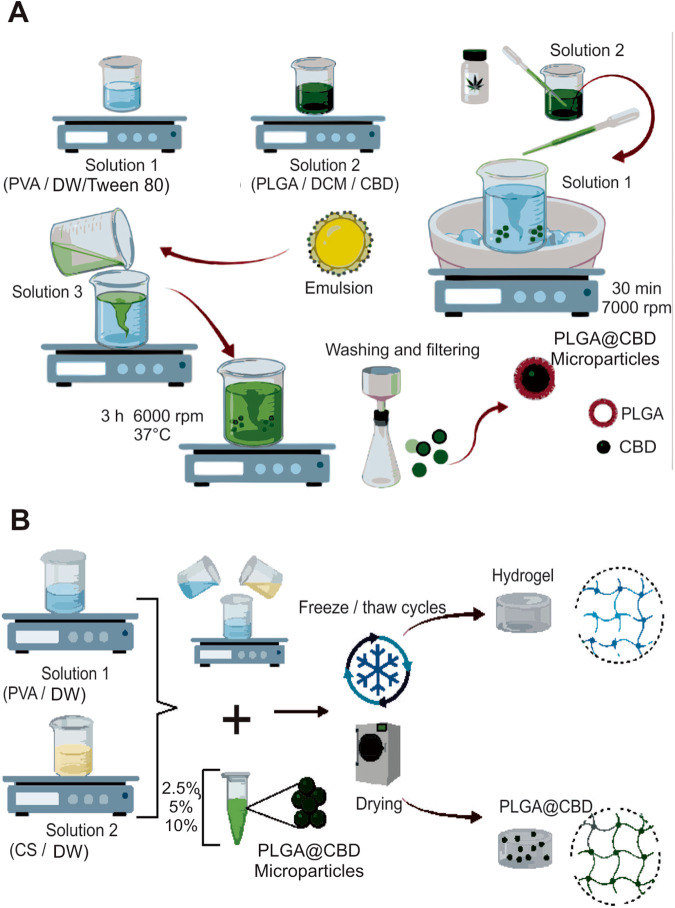
Illustration of the synthesis route of PLGA@CBD microparticles (**A**) that were subsequently embedded in a CS/PVA hydrogel (**B**). PLGA poly (lactic-*co*-glycolic acid), CBD cannabidiol, DCM dichloromethane, CS chondroitin sulfate, PVA polyvinyl alcohol

The synthesis route is illustrated in Fig. 1A. A single emulsion (oil/water) solvent evaporation method was used to prepare PLGA microspheres that contained CBD [20]. The oil/water emulsion technique was selected due to the limited solubility of CBD in water and polar organic solvents. Additionally, this method was chosen for its ability to achieve uniform particles and facilitate versatile drug inclusion [6, 20]. The first solution (water phase) of distilled water (DW), 0.5% w/v PVA was prepared. Next, the second solution (oil phase) was prepared by dissolving PLGA (0.15 g) and 300 µL of CBD in 10 mL of DCM at room temperature. This oil phase was dropped into a second solution that was kept in an ice bath. The resulting emulsion was homogenized for 30 min at 7000 rpm (Heidolph Silent Crusher M, Schwabach, Germany) at low temperature. The oil/water emulsion was added to a third solution (DW/PVA 0.5% w/v) and the system was stirred for 3 h at 6000 rpm at 37 °C. This step was conducted in an open system to allow evaporation of the organic solvent and promote solidification of the microparticles. The PLGA@CBD microparticles were collected by filtration on filter paper with a porous size of 0.5 µm and washed three times with DW to remove residual surfactants, frozen at −20 °C, lyophilized, and stored at −20 °C until use.

### Encapsulation and loading efficiencies of PLGA@CBD microparticles

The amount of CBD that was encapsulated into the PLGA microparticles was determined (*n* = 3) using a UV-Vis spectrometer (model Lambda 24; Perkin-Elmer, Waltham, MA, USA). The PLGA@CBD microparticles were completely ground and soaked in DCM for 24 h with stirring for extraction. The solutions obtained were centrifuged at 5000 rpm for 15 min and the supernatants were analyzed by fluorescence spectroscopy at an excitation wavelength of 280 nm and an emission wavelength of 307 nm. The CBD content in the supernatants was estimated using the fluorescence data and a previously constructed calibration curve (*R*^2^ > 0.999). Encapsulation efficiency (EE%) and loading capacity (Loading%) were calculated by using Eqs. (1) and (2)[34]:

1 $${\rm{EE}}\left( \% \right)=\frac{{\rm{Amount\; of\; CBD\; in\; the\; microparticles}}}{{\rm{Total\; amount\; of\; CBD\; utilized}}}\times 100,$$

2 $${\rm{Loading}}\;( \% )=\frac{{\rm{Amount}}\; {\rm{of}}\; {\rm{CBD}}\; {\rm{in}}\; {\rm{the}}\; {\rm{microparticles}}}{{\rm{Total}}\; {\rm{amount}}\; {\rm{of}}\; {\rm{microparticles}}}\times 100.$$

### Synthesis of CS/PVA and CS/PVA/PLGA@CBD hydrogels

The CS/PVA hydrogels (with and without PLGA@CBD) were synthesized using the experimental protocol described by Grazioli et al. [33]. Briefly, a CS solution (3% w/v) was prepared using DW and kept under magnetic stirring at room temperature for 1 h. In parallel, a PVA solution (1.5% w/v) was prepared by solubilizing the polymer in DW at 80 °C under stirring for 1 h. The CS and PVA solutions were blended and homogenized with magnetic stirring at room temperature for 1 h. The resulting solution was then frozen at −20 °C for 16 h and then allowed to thaw for 4 h. In total, four freezing/thawing cycles were performed for each hydrogel specimen, which was subsequently dried by lyophilization (−55 °C for 48 h). For synthesizing the CS/PVA/PLGA@CBD hydrogels, specific amounts of PLGA@CBD microparticles (2.5%, 5.0%, or 10.0% w/w) were added to the CS/PVA solution before the freeze/thawing process. The synthesized hydrogels were labeled as follows: Control hydrogel (hydrogel without PLGA@CBD microparticles); PLGA@CBD2.5% hydrogel; PLGA@CBD5.0% hydrogel; and PLGA@CBD10.0% hydrogel.

### Physicochemical characterization of the PLGA@CBD microparticles and synthesized hydrogels

#### SEM and FTIR analyses

Morphologies of the PLGA@CBD microparticles and hydrogels were observed by using a scanning electron microscope—SEM (JSM 6610LV; Jeol Ltd., Tokyo, Japan). The size of the PLGA@CBD microparticles was calculated using the Size Meter® software v.1.1, with the differentiation threshold established according to the image scale. For the hydrogels, specimens were fractured and images were obtained of their cross-sectioned portions. Before image acquisition, all specimens were sputter-coated with gold.

The chemical nature of the synthesized materials was evaluated by Fourier transform infrared spectroscopy—FTIR (IR-Affinity-1; Shimadzu) coupled to an attenuated total reflectance device. Before acquisition of the spectra, disk-shaped specimens were formed using KBr and analyzed in the range of wavelengths from 400 to 4000 cm^−1^, with 100 coadded scans and resolution of 2 cm^−1^.

#### Thermogravimetric analyses

Thermogravimetric (TG) and derivative thermogravimetry (DTG) analyses were performed using 5–6 mg of the dried specimens of control hydrogel, PLGA@CBD10% hydrogel and PLGA@CBD microparticles accurately weighed (*n* = 3). For this analysis, the hydrogels and PLGA@CBD microparticles were crushed, put into hermetically-sealed platinum sample holders and analyzed in a temperature range of 30–500 °C, under a flow of N_2_ at 50 mL/min, and 10 °C/min heating rate (SDT Q600 analyzer; TA Instruments, New Castle, DE, USA).

#### Swelling capacity

The liquid uptake property (swelling capacity) of the control hydrogel and hydrogels containing the PLGA@CBD microparticles was assessed (*n* = 3) by a conventional gravimetric procedure [2]. For this analysis, 50 mg of each type of hydrogel specimens were left in contact with phosphate buffer solution (PBS, pH 7.4) at 37 °C kept under low-speed stirring. The specimens were recovered at specific time intervals and weighed. The swelling degree at different time intervals was calculated using Eq. (3):

3 $${\rm{Swelling}}\left( \% \right)=\left(\frac{{\rm{Ws}}-{\rm{W}}{\rm{d}}}{{{\rm{W}}}_{{\rm{d}}}}\right)\times 100,$$

where *w*_*d*_ is the initial dry weight, and *w*_*s*_ is the swollen weight of the hydrogel specimens.

#### Water contact angle

The values of water contact angle formed on the surface of the synthesized hydrogels were measured (*n* = 3) by the sessile drop method [2] using an optical tensiometer (Theta Lite TL101; Biolin Scientific, Gothenburg, Sweden). Standardized 5 μL DW drops were dispensed directly onto the surface of disk-shaped specimens (diameter 10 mm, thickness 2 mm). Immediately after dispensing the drop, a dynamic reading in real time was taken of the right and left contact angles to the surface formed, using 20 frames/s for 20 s. The contact angle (θ) was recorded as the mean between the right and left readings.

#### In vitro degradation assays

In vitro degradation assays (*n* = 3) were performed in PBS (pH 7.4) medium [2]. Pre-weighed specimens of the control and PLGA@CBD10.0% hydrogels were placed in sealed vials filled with 10 mL of PBS at 37 °C. After 48 and 144 h of storage, the specimens were removed from the vials, washed with ultrapure water to remove undesired compounds, and oven-dried to a constant weight. The percentage of degradation after each storage interval was calculated using Eq. (4):

4 $${\rm{Degradation}}\left( \% \right)=\left(\frac{{{\rm{w}}}_{0}-{{\rm{w}}}_{{\rm{t}}}}{{{\rm{w}}}_{0}}\right)\times 100,$$

where *w*_*0*_ is the specimen initial weight, and *w*_*t*_ is the weight after soaking.

#### Total porosity

The total porosity of the hydrogels was evaluated by the conventional liquid displacement method [2]. Cube-shaped specimens (*n* = 3) of control and PLGA@CBD10.0% hydrogels were immersed in a known initial volume (*v1*) of acetone at room temperature for 24 h. After impregnation with acetone, the volume of the hydrogel-acetone set (*v2*) was determined. Finally, the acetone-impregnated specimen was separated and the remaining liquid volume was determined (*v3*). The total porosity was calculated using Eq. (5):

5 $${\rm{Total\; porosity}}\left( \% \right)=\frac{({\rm{v}}1-{\rm{v}}3)}{({\rm{v}}2-{\rm{v}}3)}\times 100.$$

#### Mechanical properties

Cubic specimens (1 mm^3^) of the control and PLGA@CBD10.0% hydrogels were swelled up to equilibrium in PBS [2]. Next, the swollen specimens were mechanically compressed using a texture analyzer (TA XT plus C; Stable Micro Systems, London, UK) operating with 50% maximum strain, 0.5 mm/s test speed, and a 50 N load cell. The response-variables were Young’s modulus, rupture force, and strain at rupture (*n* = 3).

### Biological properties

#### Cell viability assay

Primary human dental pulp cells (hDPCs) were obtained, cryopreserved, and characterized, as reported in a previous study [33]. Previous cell characterization was performed by flow cytometry, with CD73, CD90, and CD105 as positive markers and CD34 and CD45 as negative markers (BD Biosciences, San Jose, CA, USA). The cells were resuspended in PBS and incubated for 30 min at room temperature (protected from light) with specific antibodies labeled with fluorescein isothiocyanate or phycoerythrin. Cells were then washed with PBS and analyzed with a flow cytometer (FACS Calibur; BD Biosciences).

Biocompatibility of the synthesized hydrogels were determined in a cell viability assay using (3-(4,5-dimethylthiazol2-yl)-2,5-diphenyltetrazolium bromide)—MTT, according to ISO standard 10993-5 [35]. Disk-shaped hydrogel specimens (diameter 5 mm, thickness 1 mm thickness, *n* = 3 *per* group) and PLGA@CBD microparticles were prepared and sterilized by UV treatment (30 min on each side). The specimens were placed in sterile glass vials containing 5 mL of alpha minimal essential medium supplemented with 10.0% fetal bovine serum, L-glutamine, and 1% penicillin-streptomycin, then incubated at 37 °C. At predetermined time points spanning up to 21 days [23, 36], 500 μL aliquots of the medium released by the various hydrogels and microparticles were collected. Equal amounts were then added to each vial to ensure the maintenance of the initial volume at a constant level.

The hDPCs were seeded in 96-well plates at a rate of 1 × 10^4^ cells per well and allowed to adhere at 37 °C for 24 h, with 5% CO_2_. The cell culture media were subsequently replaced with culture media containing the eluates previously collected at different time intervals (1, 2, 7, 21 days), which had been in contact with the disk-shaped specimens. This mixture was left undisturbed for an additional 24 h. The solutions in each well were then removed and replaced with 200 µL of MTT solution (5 mg/mL). After 4 h incubation at 37 °C in the dark, the MTT solution was removed and 200 μL dimethyl sulfoxide was added to each well, and the plates were placed on a shaker for 5 min. The absorbance at 540 nm was determined using a spectrophotometer (TP-Reader; Thermoplate, Shenzhen, China). Cell culture experiments were conducted in triplicate, considering specific time intervals: 1, 2, 7, and 21 days. The selection of these time intervals was based on previous assessments of a potential drug delivery system, taking into consideration the higher degradation rate of PLGA [6].

#### Antimicrobial assay—Direct contact test

The direct contact test was performed to investigate the antimicrobial effect of the hydrogels in a planktonic model [36, 37]. The hydrogels were tested against *Staphylococcus aureus* (ATCC 25923). First, a pilot direct contact test was carried out with the control hydrogel, PLGA@CBD10.0% hydrogel and the PLGA@CBD microparticles to determine inhibition halos (12.03 ± 2.1 mm). Subsequently, the antimicrobial effect was determined considering the hydrogel degradation over time and hydrophobic nature of CBD. Dry specimens (200 mg) of the control, PLGA@CBD2.5%, PLGA@CBD5.0%, and PLGA@CBD10.0% hydrogels (*n* = 3 *per* group) were disinfected by UV radiation at 37 °C for 30 min. In addition, an amount of PLGA@CBD microparticles equivalent to the amount embedded in the PLGA@CBD10.0% hydrogel was separated and disinfected. Next, the specimens were individually incubated in glass vials with 5 mL of sterile PBS at 37 °C. At default time intervals up to 21 days, 500 μL aliquots were drawn and replaced with an equivalent amount of fresh PBS. The aliquots retrieved were stored at -20 °C until further use. Each bacterial plate was divided into four zones, namely 10 μL of 2% chlorhexidine digluconate (CHX; positive control), 10 μL of DW (negative control), and 20 μL of the hydrogel aliquots (two zones/plate randomly assigned). After 24 h of incubation, the diameters (in mm) of the clear zones of growth inhibition were measured.

### Statistical analysis

For all response-variables, data were tested for normality using Shapiro-Wilk and equal variance tests. Swelling capacity and water contact angle data were submitted to one-way Analysis of Variance—ANOVA. In vitro degradation assay, cytotoxicity, and antimicrobial activity data were analyzed using Two-Way ANOVA (different hydrogels *vs*. time). Porosity data were analyzed using the Student’s*-t* test. All pairwise multiple comparisons were carried out using Tukey’s *post-hoc* test. Young’s modulus of elasticity, rupture force and strain at rupture were compared with use of the Mann-Whitney rank sum test. For all tests, a significance level of *α* = 0.05 was used. The statistical analyses were carried out using SigmaPlot 12.0 software (Systat Inc., San Jose, CA, USA).

## Results

### Characterization of the PLGA@CBD microparticles and hydrogels

**Fig. 2:**
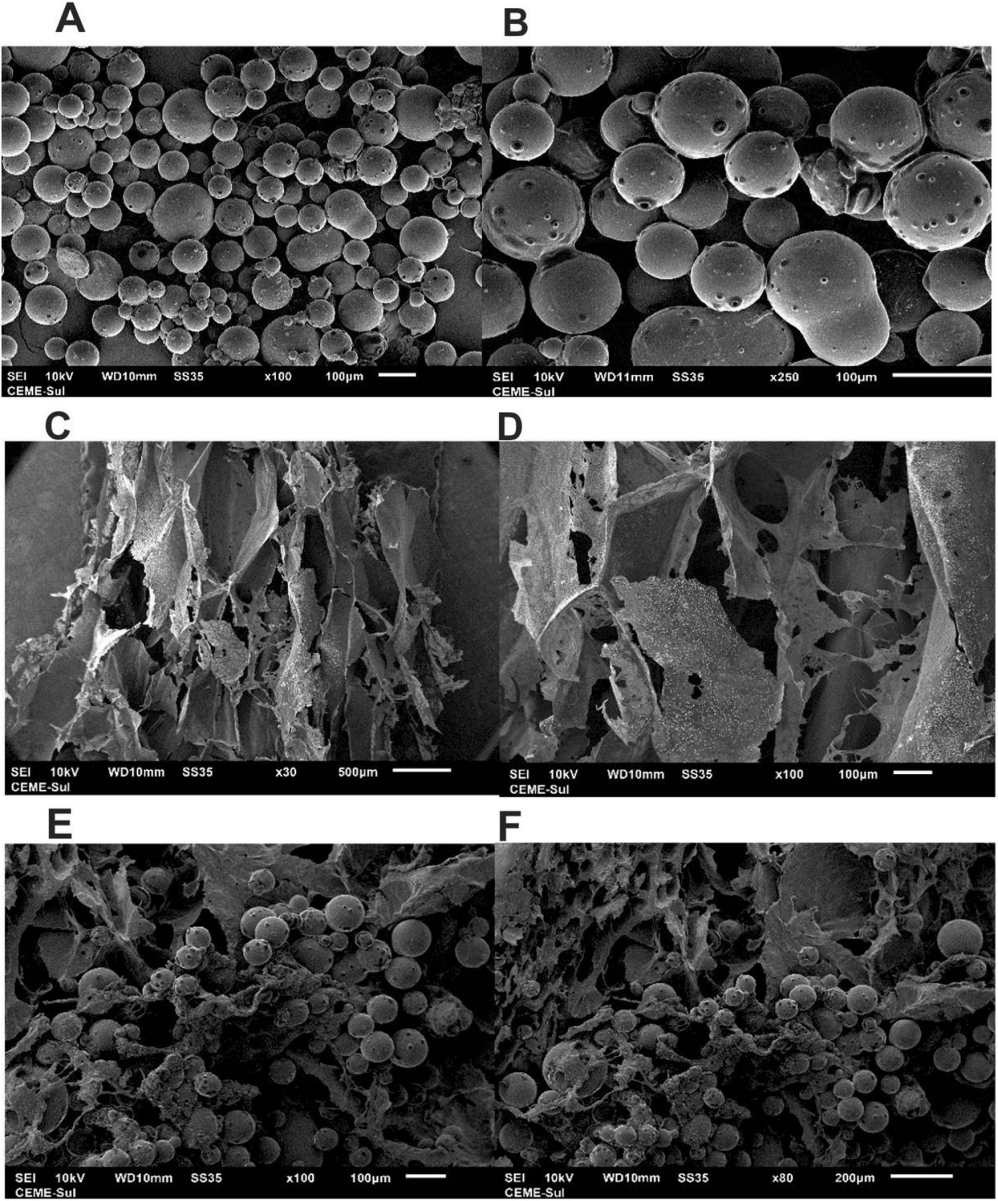
SEM images of PLGA@CBD microparticles alone (**A**, **B**), control hydrogel (**C**, **D**), PLGA@CBD10.0% hydrogel (**E**), and PLGA@CBD5.0% hydrogel (**F**)

Images of the PLGA@CBD microparticles obtained using SEM (Fig. 2A, B) demonstrated a spherical shape and narrow range of size distribution (81 ± 8 µm). At high magnification, the microparticles showed a smooth and dense surface. Overall, the EE% was computed to be around 52 ± 0.3%, while the Loading% was 50 ± 1.1%. The control hydrogel exhibited an irregular morphology with pores that were heterogeneously distributed (Fig. 2C, D). Moreover, there was interconnectivity among the pores of different sizes, which is typical for hydrogels prepared by using the freeze-thaw method [20]. Images obtained of the PLGA@CDB5.0% and PLGA@CDB10.0% hydrogels (Fig. 2E, F) revealed that the typical porous and irregular morphology was preserved and microparticles were uniformly distributed on the walls of the pores. Comparatively, there were no noticeable differences observed in the morphology and size of the microparticles before and after being embedded in the hydrogel matrices.

**Fig. 3:**
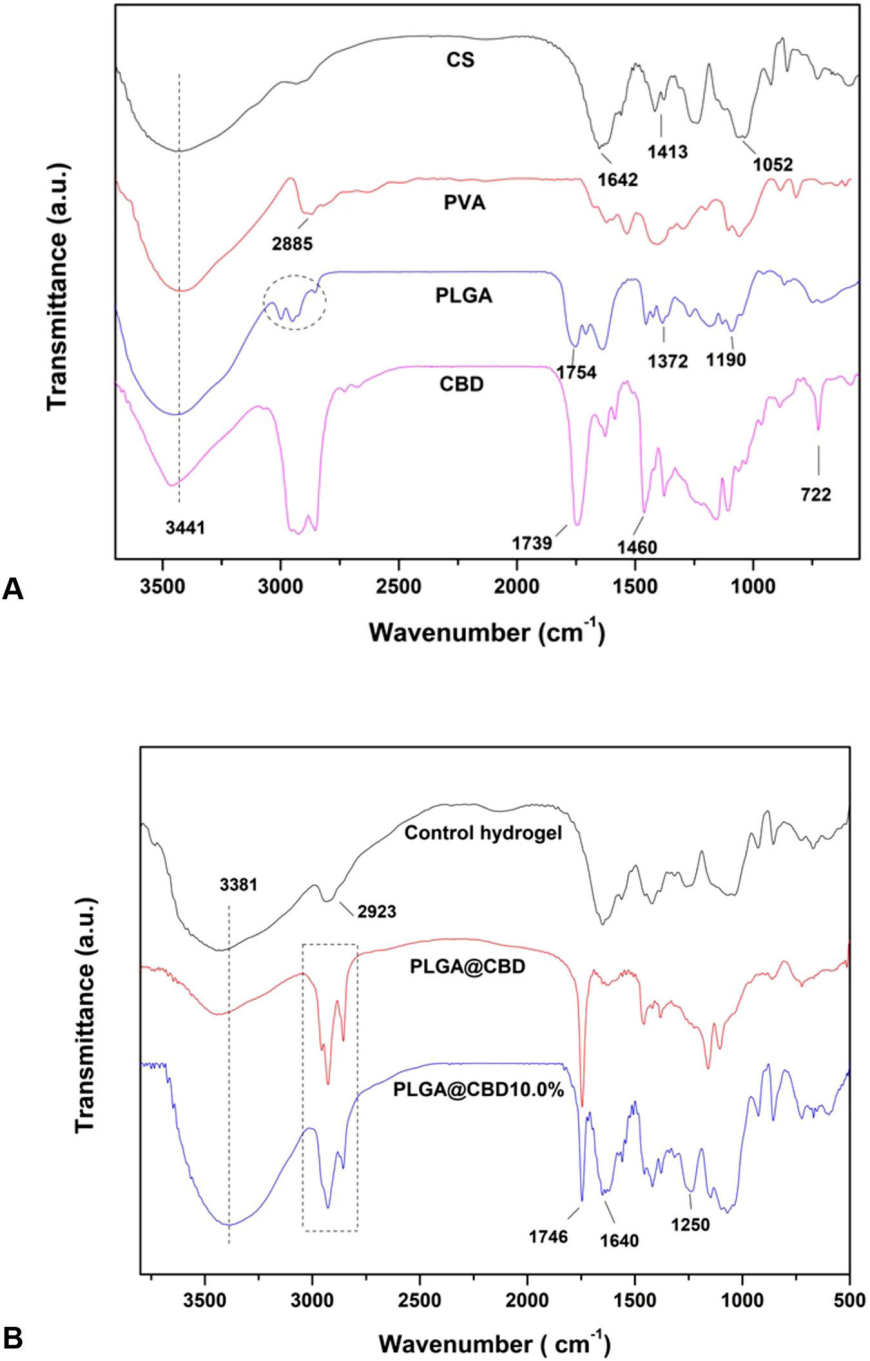
**A** FTIR spectra of the precursors CS, PVA, PLGA, and CBD. **B** FTIR spectra of PLGA@CBD microparticles, control hydrogel, and PLGA@CBD10.0% hydrogel

The FTIR spectra obtained from the precursor materials utilized for the synthesis of the PLGA@CBD microparticles and the control and PLGA@CBD10% hydrogels were in accordance with the previous literature [2, 3, 33] (Fig. 3A). For PLGA@CBD microparticles, characteristic bands were observed at around 3450 cm^−1^ (O-H stretch) 2850–3000 cm^−1^ (C-H stretch), and 1754 cm^−1^ (C=O stretch) due to PLGA. Furthermore, bands at around 3500 cm^−1^ (O-H stretch), 2987 cm^−1^ (C-H_arom_ stretch), 1746 cm^−1^ (C=O stretch, ester of fatty acid), and 1460 cm^−1^ (C-H scissor) were attributable to CBD (Fig. 3B). The FTIR spectra of the synthesized hydrogels (control and PLGA@CBD10.0%) exhibited the main bands assigned to the polymers (CS and PVA) with some shifting. Characteristic bands were observed at around 3381 cm^−1^ (O-H stretch), 2900 cm^−1^ (C-H stretch), 1642 cm^−1^ (C=O stretch), 1250 cm^-1^ (S=O stretch), and 1052 cm^−1^ (C-O-C stretch). In addition to the bands of CS and PVA, the FTIR spectrum of the PLGA@CBD10.0% hydrogel showed bands proceeding from PLGA@CBD microparticles as seen in Fig. 3B.

**Fig. 4:**
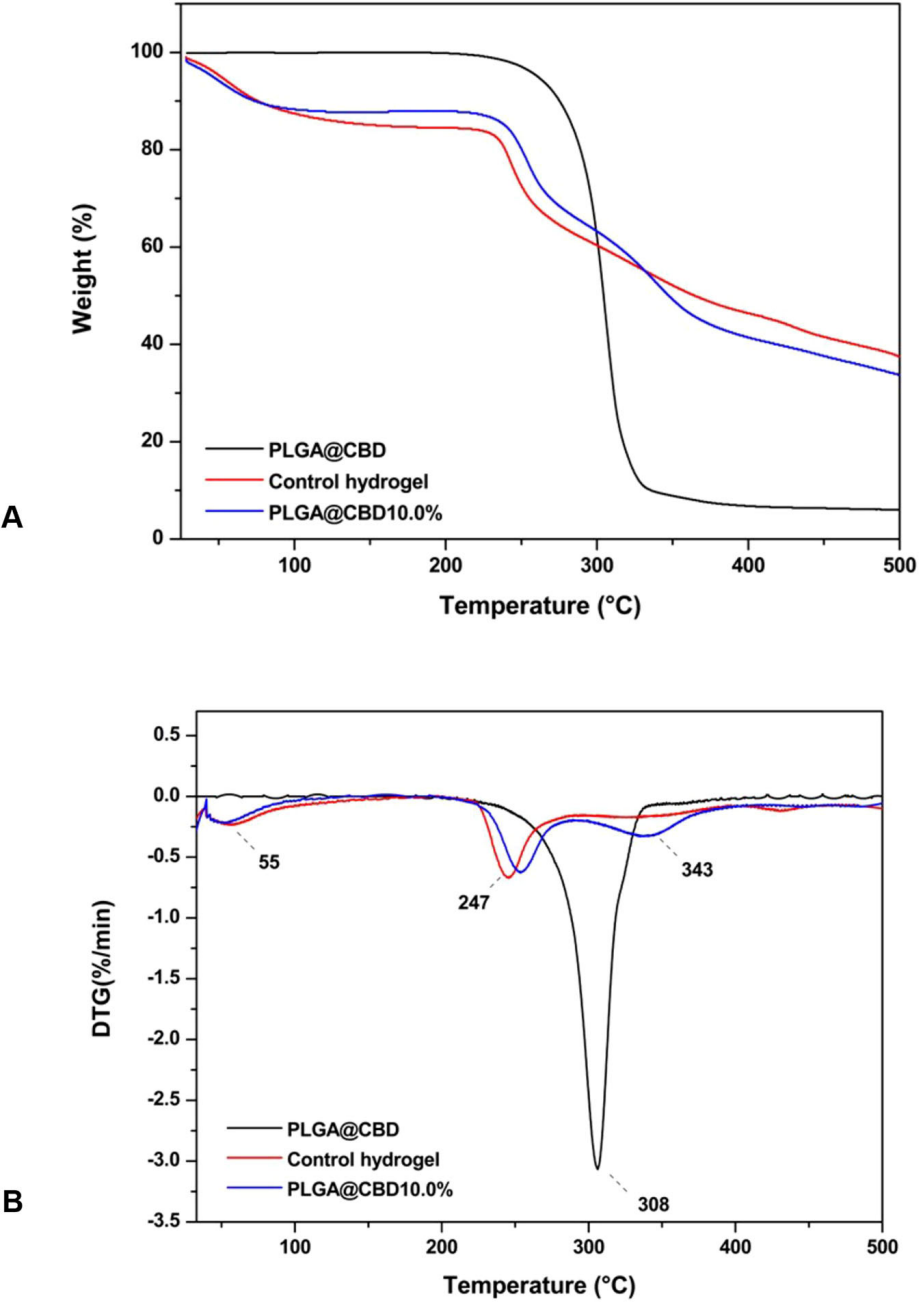
**A** TG and (**B**) DTG curves of the PLGA@CBD microparticles, control hydrogel, and PLGA@CBD10.0% hydrogel

Results for the thermal analyses are shown in Fig. 4A, B. TG/DTG curves of PLGA@CBD microparticles showed only one weight-loss stage with a maximum temperature of 308 °C attributable to the depolymerization of the PLGA matrix and degradation of the CBD oil. The TG curve of the control hydrogel exhibited two weight-loss stages, where the first (from 30 to 100 °C) resulted in a 12% loss due to water. The second, from 220 to 260 °C (25% weight-loss) was due to thermal decomposition of the polymers used for synthesis of the hydrogel. The TG/DTG curves of PLGA@CBD10.0% hydrogel revealed the appearance of one additional weight-loss stage (from 260 to 380 °C), which resulted in a 25% loss.

Results relative to maximum swelling degree and water contact angle computed for the control hydrogel and the hydrogels embedded with 2.5%, 5.0%, and 10.0% PLGA@CBD microparticles are presented in Table 1. Overall, all hydrogel specimens showed rapid swelling kinetics and achieved the condition of equilibrium at around 40 min after being in contact with PBS. The degree of swelling was reduced with the addition of PLGA@CBD microparticles. However, the hydrogels had similar water contact angles (*p* = 0.568), thus establishing a similar hydrophilic behavior.

**Table 1:** Means (standard deviations) for maximum swelling degree and water contact angle computed from the synthesized hydrogels, *n* = 3

Hydrogel	Swelling (%)	Water contact angle (°)
Control	490 (2)^A^	65.7 (16.5)^A^
PLGA@CBD2.5%	311 (1)^B^	57.1 (17.1)^A^
PLGA@CBD5.0%	311 (10)^B^	50.1 (13.1)^A^
PLGA@CBD10.0%	351 (9)^C^	50.2 (12.7)^A^

Capital letters in the same column indicate significant differences between hydrogels (p < 0.05).

Results of the in vitro degradation test in PBS and total porosity are shown in Table 2. The highest percentage of degradation was observed at 144 h for control hydrogel and PLGA@CBD10.0% hydrogel, which demonstrated a similar behavior according to statistical analysis (*p* = 0.122). The PLGA@CBD10.0% hydrogel showed a lower percentage of total porosity compared with the control hydrogel (*p* < 0.001). Table 3 shows the results of the mechanical properties. The rupture force and maximum strain at rupture were higher in the PLGA@CBD10.0% hydrogel (*p* = 0.05) compared with the control, whereas the Young’s modulus of elasticity was similar between them (*p* = 1.0).

**Table 2:** Means (standard deviations) for the in vitro degradation in PBS at different times and porosity, *n* = 3

Hydrogel	Degradation (%)		Total porosity (%)
	48 h	144 h	
Control	54.5 (2.4)^A,a^	65.0 (7.9)^A,b^	66.6 (0.2)^A^
PLGA@CBD10.0%	58.3 (2.0)^A,a^	65.9 (3.4)^A,a^	50.0 (0.1)^B^

Capital letters in the same column indicate significant differences between hydrogels; lowercase letters in the same line indicate significant differences between times (*p* < 0.05).

**Table 3:** Medians (Q1–Q3) for Young’s modulus, rupture force, and maximum strain at rupture (*n* = 5)

Hydrogel	Young’s modulus, MPa	Rupture force, *N*	Maximum strain at rupture, %
Control	0.10 (−0.19–0.70)^a^	40 (37–47)^a^	60 (58–66)^a^
PLGA@CBD10.0%	0.12 (0.08–0.52)^a^	103 (99–111)^b^	91 (90–94)^b^

Lowercase letters in the same column indicate significant differences between hydrogels (*p* < 0.05)

### Cell viability assay

**Fig. 5:**
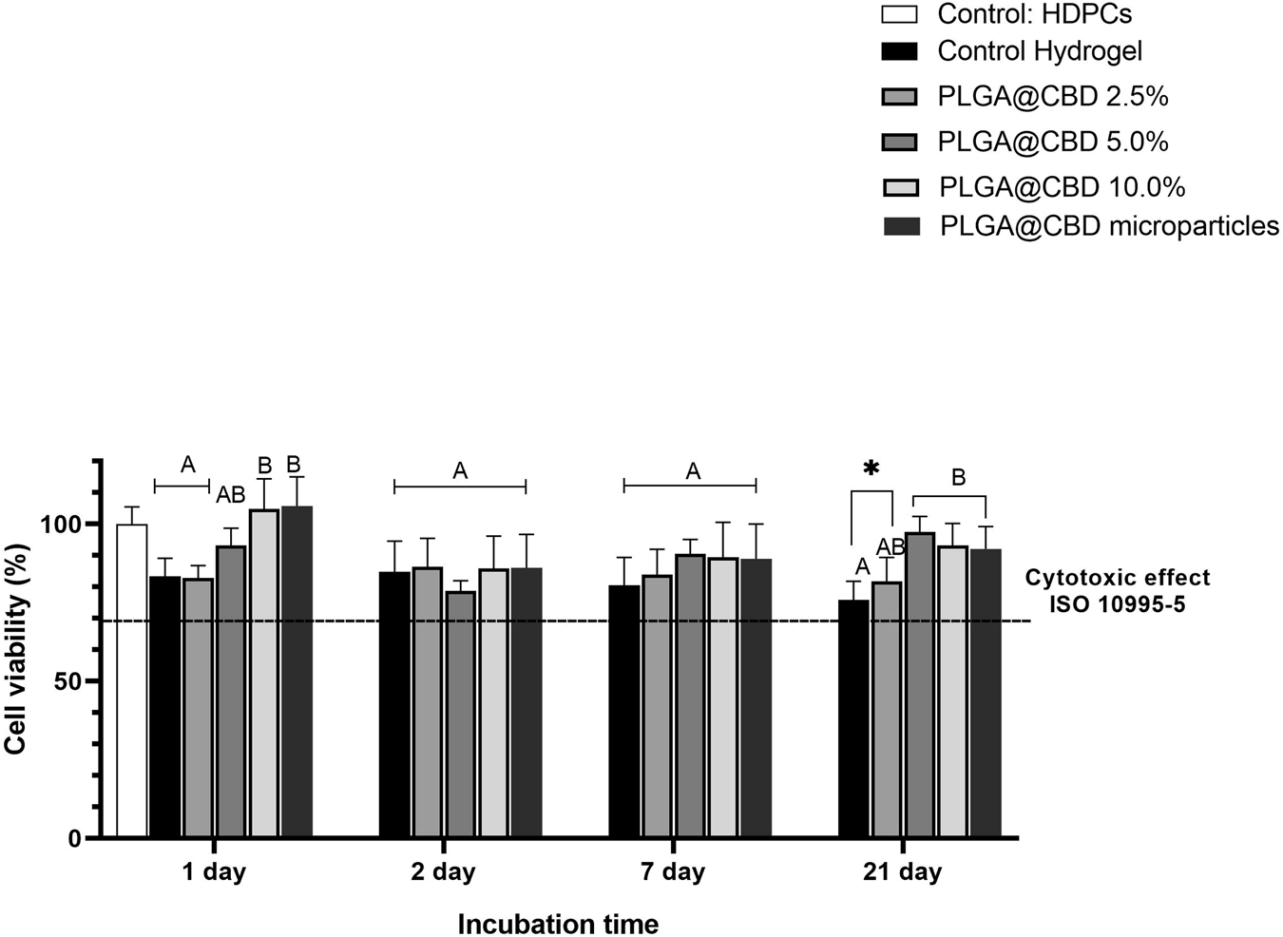
The cell assay quantified the viability (%) of human dental pulp cells (hDPCs) in response to aliquots (previously collected on days 1, 2, 7, and 21) of the control hydrogel, PLGA@CBD hydrogels (2.5–10.0%), and PLGA@CBD microparticles (*n* = 3) over a 24-h period. Statistical analyses were conducted to compare groups within the same day. Distinct letters indicate statistically significant differences between groups. The asterisk indicates significant differences between the groups and the cell control. The dotted line corresponds to the limit on toxic effects according to the standard ISO 10993-5

Cell viability results are shown in Fig. 5. The control hydrogel, PLGA@CBD hydrogels, and PLGA@CBD microparticles evaluated showed similar values compared with the cell control. When comparing the percentages of cell viability over time, the groups PLGA@CBD5.0% hydrogel, PLGA@CBD10.0% hydrogel and PLGA@CBD microparticles showed differences on the first day (*p* < 0.001), presenting the highest cell viability when compared with the other groups. At 21 days, the control hydrogel (*p* = 0.003) and PLGA@CBD2.5% hydrogel (*p* = 0.01) showed lower values than the cell control and other hydrogels (*p* < 0.001). Subsequently, the number of surviving cells decreased in aliquots of all hydrogels collected on day 2. However, the percentages of cell viability were similar among groups on day 2 (*p* = 0.122) and day 7 (*p* = 0.192).

### Antimicrobial activity

**Fig. 6:**
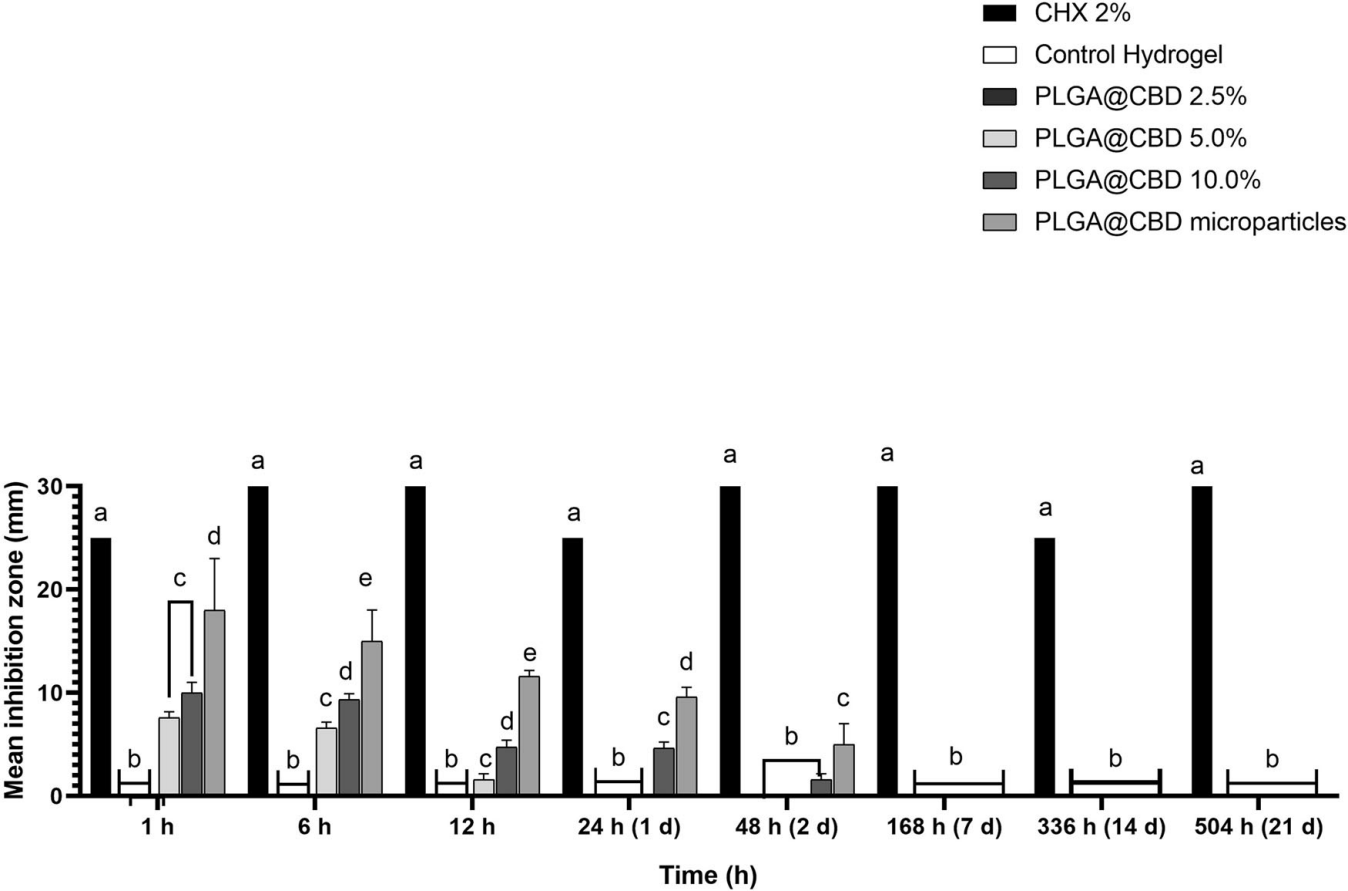
Results of the agar diffusion assays are represented as a mean inhibition zone (mm) against the *S. Aureus* in response to aliquots on 1, 6, 12, 24 and 48 h, 7, 14 and 21 days of control hydrogel, PLGA@CBD (2.5–10.0%) hydrogels, and PLGA@CBD microparticles. Statistical analyses compared groups within the same time. Distinct letters indicate statistically significant differences between groups compared with chlorhexidine (CHX) control

Figure 6 shows results of the agar diffusion assays (antimicrobial activity). The control and PLGA@CBD2.5% hydrogels showed no bacterial inhibition at all time points. The PLGA@CBD5.0% and PLGA@CBD10.0% hydrogels, and the PLGA@CBD microparticles, showed increased inhibition zones in the first hour of contact when compared with the subsequent times. After the first hour, the inhibition zones decreased (dose-time response) until the first 48 h of contact. Higher concentrations of PLGA@CBD microparticles in the hydrogels were associated with increased antimicrobial activity. All groups at all times showed differences in relation to the chlorhexidine control (*p* < 0.001).

## Discussion

Due to its chemistry, pharmacology, and molecular targets, including CB1 and CB2 receptors and other components of the endocannabinoid system, CBD may be an important mediator of the tissue healing process [25, 38–40]. Thus, we demonstrated the successful loading of CBD oil in PLGA microparticles and their embedment in a CS/PVA hydrogel matrix, which may serve as a scaffold material for biomedical applications in tissue engineering, e.g., bone and tissue repair. This strategy offers conditions to overcome the inherent limitations of using CBD oil by itself and enhances its applicability in specific sites.

CBD was loaded in PLGA microparticles. PLGA was selected because this polymer has loading capacity for hydrophobic and hydrophilic molecules, as it is biocompatible, biodegradable, and has the ability to perform sustained release and protect the drug against initial degradation [38]. After the synthesis of the PLGA@CBD microparticles via an o/w emulsion technique, the EE% was evaluated to establish the amount of oil that was actually associated with the particles, in relation to the total quantity of drug present in the formulation [41]. These data have a great influence on the release rate of the drug [42]. The EE% result was compatible with that of the single solvent emulsion technique [43]. In the case of encapsulation of substances such as oils and extracts in large quantities, the EE% of the microparticles could be reduced [19, 44]. One option to increase this percentage would be to reduce the size to a nanometric scale, and in turn, increase the bioavailability and load capacity [6, 30]. The morphological and textural analyses demonstrated that the microparticles have a typical spherical shape, which has also been observed by other authors who prepared PLGA microparticles using a similar technique [43]. Images obtained by SEM confirmed the embedment of the microparticles in the PLGA@CBD hydrogels. As seen, the spherical shape of the microparticles in the hydrogels was maintained, confirming the effectiveness of the methodology [43].

Overall, the microparticles showed good distribution throughout the hydrogel matrices even at the highest amount tested (10.0% w/w), which indicated compatibility between the microparticles and the polymers. Indeed, the broadening of the band associated with the O-H bond (between 3600 and 3000 cm^−1^) in the FTIR spectrum of PLGA@CBD10.0% compared with the spectrum of the control hydrogel suggested the increase in hydrogen bonds caused by interactions between the hydrogel matrix and microparticles. The oxygen-rich functional groups of PLGA interacted with hydrogen atoms of the CS/PVA matrix, benefiting the distribution of the microparticles and increasing their stabilization within the hydrogel [38]. Another aspect triggered by this interaction was observed in the TG/DTG data. The weight-loss associated with water evaporation (i.e., dehydration) was lower for PLGA@CBD10.0% probably because this material contained a lower amount of free water compared with the control hydrogel. Due to the partial consumption of hydrophilic groups of the hydrogel matrix caused by interactions with the PLGA@CBD microparticles, the fraction of water molecules in this hydrogel was lower. This behavior also impaired the swelling capacity of the PLGA@CBD hydrogels compared with the control. As seen in Table 1, the presence of the PLGA@CBD microparticles in the hydrogel matrix decreased the swelling ability of these materials. The trend varied according to the amount of microparticles used. Noticeably, the wettability of the PLGA@CBD hydrogels did not undergo changes when compared with the control, probably because the majority of the microparticles preferentially remained in the bulk phase of the materials, thus exerting a minimal effect on their surface.

In contrast, interactions between the microparticles and CS/PVA matrix arose as additional forces that may hold the polymer chains together in a manner similar to that of crosslink points. This suggestion corroborated the increased thermal stability observed for PLGA@CBD10.0% compared with the control hydrogel [6]. It is relevant to note that loading CBD into the PLGA microparticles also increased the thermal stability of the oil, which has a boiling point starting at around 170 °C [44, 45]. An additional observation relative to the increase in crosslinking points was the reduction of the total porosity calculated for PLGA@CBD10.0% compared with the control hydrogel (Table 1). Lower porosity also reduced the swelling ability of hydrogels, as demonstrated in the literature [46]. However, the PLGA@CBD hydrogels maintained a high porosity (>50%) providing adequate fluid flow to supply nutrients, bioactive compounds, growth factors and ensure the removal of the degradation by-products [39, 47].

Together with the thermal and porosity properties and increase in crosslinking points, the mechanical properties of the PLGA@CBD hydrogels were superior to those computed for the control hydrogel. Despite causing a low effect on the elasticity of hydrogel, the presence of PLGA@CBD microparticles seemed to favor elasticity and elastic deformation of the PLGA@CBD10.0% hydrogel, helping to improve distribution of mechanical stresses during the compression test [31]. The mechanical properties exhibited by the synthesized hydrogels containing PLGA@CBD microparticles were favorable in relation to their deformation, so that they were comparable with different types of scaffolds described in other investigations, despite the differences in composition [2, 31, 32, 48].

Once the embedment of PLGA@CBD microparticles in the CS/PVA hydrogel was confirmed, the in vitro stability of the biomaterial prepared was evaluated. Degradation is a crucial property needed for a potential polymeric scaffold [2] since it controls its long-term performance and regulates the rate of cell growth. In bone or tissue repair, the scaffold acts as mechanical/structural support while new cells/tissues regenerate the area treated [2, 3]. In this study, the scaffold carried CBD-loaded microparticles, therefore, its stability could be an indirect estimator of bioavailability of this compound, together with its release and diffusion characteristics [47]. The in vitro degradation was tested only for the control hydrogel and the hydrogel with 10.0% PLGA@CBD microparticles because they were the hydrogels with the highest contrast in composition and, thus, more prone to show differences in their structure and morphology. The in vitro degradation was evaluated in the first 48 h, with percentages of degradation higher than 50%, but the highest percentage of degradation was up to 144 h (7 days). Degradation was comparable in both hydrogels. The degradation rate observed could provide the time necessary for primary proliferation, migration, and cell adhesion [44, 46, 49]. This may favor the action of CBD in increasing cellular migration dose-dependently in the first hours of contact with cell lineages similar to those of human osteoblasts [28].

The results of the present investigation differed from those reported by another study with similar control hydrogel [50, 51] or other hydrogels with microparticles of PLGA and CBD with different chemical elements [20, 23]. In those studies, a longer degradation rate was observed. Different crosslinking between hydrogel contents and an increase in their density, among other factors, may prolong the degradation rate of hydrogels [18]. The discrepancy observed may be explained by the composition of the hydrogels: Kamali et al. [20] used CBD-PLGA microspheres in a gelatin/nanohydroxyapatite scaffold different from the one used in the present research. Other factors related to the PLGA@CBD microparticles could modify the rate of degradation, such as the ratio of the components of the PLGA copolymer, the order of the monomers, end groups, molecular weight [6, 38], and the diameter or size of the microparticles [6, 38, 51, 52]. The hydrophobic nature of CBD and its prolonged degradation [11] may change the degradation mechanism from bulk erosion to PLGA surface deterioration, hydrolysis, and polymer matrix degradation [38]. Relative to hydrophilicity, all hydrogels analyzed showed a rapid degree of swelling and reached stability after 40 min of immersion, but the control hydrogel showed a higher maximum degree of swelling compared with all the PLGA@CBD-loaded hydrogels. This result suggested that the control hydrogel and PLGA@CBD hydrogels had a considerable liquid retention capacity. Moreover, it confirmed their hydrophilic nature, which may decrease with the presence of PLGA@CBD microparticles probably due to the highly hydrophobic nature of CBD [8, 31, 47]. However, this characteristic did not seem to affect the water contact angle and wettability though; the modified and unmodified hydrogels showed similar diffusion of liquids.

The MTT cell viability assay was used to evaluate the biocompatibility of CBD under in vitro conditions using hDPCs. No significant changes were observed in relation to the cell control group. CBD demonstrated a dose-dependent change in cell proliferation of hDPCs and, in the range of 0.1 to 100 µmol/L, it was not cytotoxic [27]. In solutions prepared with pure CBD at low concentrations ranging from 3.0 to 9.0 µmol/L or slightly higher [20, 53], cell viability showed a significant increase. Notably, CBD at a concentration of 5 µmol/L demonstrated a positive biphasic effect on hDPCs in vitro [27], potentially directly stimulating cell migration [27, 53]. The concentrations of CBD examined exhibited a positive influence on the cell viability of hDPCs. These are cells that have a high proliferative potential and the ability to differentiate into various types of other cells such as mesenchymal cells, including chondrocytes, cardiomyocytes, adipose cells, muscle cells, osteoblasts, and odontoblasts [25, 27]. CBD may perhaps be a usable substance in tissue regeneration by promoting an important biological response in tissue healing, a complicated biological process with an initial inflammatory response followed by recruitment and differentiation of undifferentiated mesenchymal cells [20]. The data presented in this study suggested that PLGA@CBD microparticles were safe for hDPCs in vitro. Lack of signs of toxicity and viability percentages similar to the cell control in all time points indicated that PLGA@CBD hydrogels and PLGA@CBD microparticles were promising for use in biomedical applications and the potential use of CBD in the concentrations tested. However, other studies have established that higher concentrations than those applied in the present investigation may have an inverse effect and CBD could inhibit the proliferation of undifferentiated mesenchymal cells with concentration-dependent characteristics [17, 27].

Furthermore, the results of MTT analysis determined that the high percentages on the first day may be a consequence of the rapid in vitro degradation of PLGA@CBD hydrogel in the first 48 h, leaving the PLGA@CBD microparticles exposed, and causing an early release of CBD. Moreover, the microparticles made with PLGA may have a rapid initial release that could accumulate in the first days, subsequently decreasing cellular capacity and having a lag time [6]. Another study [20] showed that the in vitro release profile of CBD-loaded into particles with PLGA polymer decreased its concentration over time with the gradual release of CBD for up to 25 days [20]. This suggested the belief that increasing the percentage of PLGA@CBD microparticles in hold hydrogels would be viable.

The results of the antimicrobial assay against *S. aureus* indicated inhibition activity, as shown by the agar diffusion test (Fig. 6). Only the aliquots collected (24–48 h) of the highest concentrations of PLGA@CBD5.0% hydrogel, PLGA@CBD10.0% hydrogel, and PLGA@CBD microparticles showed increasing zones of inhibition that could be considered an inhibitory effect. These results suggested that the inclusion of PLGA@CBD had a dose-dependent antimicrobial effect that could work for up to 48 h after release. Various studies [13, 22, 54–56] have established the antimicrobial potential that CBD could develop in low concentrations (3.2–15.9 µmol/L). Similarly, the results mirrored the quantified inhibition zones observed in other orally administered products containing CBD, as assessed through an agar diffusion test [22]. CBD reacts rapidly by disrupting bacterial cytoplasmic membranes, inhibiting the synthesis of proteins, DNA, RNA, and peptidoglycans [13, 22, 23]. Moreover, it has been suggested that PLGA@CBD hydrogels and PLGA@CBD microparticles may be effective means of delivering CBD and preventing bacterial infections, as high levels of CBD alone in suitable and compatible vehicles may create this antibacterial effect [22]. In this sense, the PLGA@CBD10.0% hydrogel could have an effective antibacterial effect against *S. aureus*, the most frequent etiological agent in infections in scarring processes. Furthermore, they can cause recurrent and chronic infections that delay healing processes due to their internalization in precursor cells, osteoblasts, and peripheral cells [57].

The present study had limitations, such as not having evaluated degradation of the particles and hydrogels mediated by enzymes. The size of the microparticles also could be smaller and thus generate greater encapsulation efficiency and influence the release of CBD after hydrolysis. However, despite the initial tendency of PLGA@CBD microparticles to aggregate on each other and the lack of quantification of the actual dose released after hydrogel degradation, we successfully synthesized PLGA@CBD microparticles from a hydrophobic substance. Subsequently, we embedded them in a hydrogel to form a stable matrix without toxicity and with an inhibitory effect on *S. aureus* strains. Further studies should investigate whether the PLGA@CBD microparticles and the inclusion in the hydrogel would be effective in the next stages of the research that include the evaluation and behavior of the modified hydrogels as possible agents for tissue healing and repair. This effect could make this potential biomedical material a successful scaffold for tissue regeneration with influence on cell behavior. This biomaterial could act as a support for tissue/cellular regeneration and be provided with adjuvant CBD microparticles [1]. Future studies on the subject could evaluate the minimum and maximum doses after the degradation of CBD released, with cell proliferation capacities and the effect on the differentiation of different cell lines to osteoblasts. In addition, the in vivo behavior of hydrogels containing CBD could be evaluated.

## Conclusions

The PLGA microparticles containing CBD oil were fabricated and successfully embedded in a CS/PVA hydrogel matrix. Despite the hydrophobic nature of CBD, the physicochemical and morphological properties were similar for the hydrogels with and without the CBD-loaded microparticles. The chemical affinity between the PLGA and the CS/PVA polymers assured the good distribution and stabilization of the microparticles in the hydrogel. As assessed, the hydrogels embedded with the microparticles presented porosity, swelling, mechanical, and in vitro degradation properties adequate for scaffolds usable for biomedical, regenerative functions. In addition, the hydrogels embedded with PLGA@CBD microparticles up to 10.0% (w/w) demonstrated an inhibitory effect against *S. aureus* without exhibiting cytotoxicity against undifferentiated cells. Taken together, the data reported in this study demonstrated that this original biomaterial loaded with CBD oil has characteristics that would enable it to be a potentially successful scaffold to support and stimulate tissue/cellular regeneration.

## Supplementary Information

Supporting Information

## Data Availability

Data presented in this article are available upon request.
