# Supplementary material for: Cannabidiol-loaded microparticles embedded in a porous hydrogel matrix for biomedical applications
Source: J Mater Sci Mater Med. 2024 Feb 14;35(1):14. doi: 10.1007/s10856-023-06773-9 (PMC10866797; doi:10.1007/s10856-023-06773-9)
Supplement: Supplementary file 1 — Supporting Information [file 10856_2023_6773_MOESM1_ESM.docx]

**Supporting Information**

**Cannabidiol-loaded microparticles embedded in a porous hydrogel matrix for biomedical applications**

Carla David ^a,b^, , Jaqueline F. de Souza ^c^, Adriana F. Silva ^b^, Guillermo Grazioli ^d^, Andressa S. Barboza ^b^, Rafael G. Lund ^b^, André R. Fajardo ^c^, Rafael R. Moraes ^b*^

^a^ Biopathological Research Group, Faculty of Dentistry (GIBFO), University of the Andes, Mérida, Venezuela

^b^ Graduate Program in Dentistry, Universidade Federal de Pelotas, Pelotas, Brazil

^c^ Laboratory of Technology and Development of Composites and Polymeric Materials – LaCoPol, Universidade Federal de Pelotas, Pelotas, Brazil

^d^ Department of Dental Materials, Universidad de la República, Montevideo, Uruguay

**Fig. S1** Result of analysis of 10 µL of 5% CBD oil with a gas chromatograph coupled to a mass spectrophotometer.
